# Case study of inferior adrenal artery as an extrahepatic collateral arterial supply to hepatocellular carcinoma

**DOI:** 10.1016/j.radcr.2024.10.026

**Published:** 2024-10-15

**Authors:** Charles Rash, Eleanor Lee, Spencer Klinsky, Wesley Angel

**Affiliations:** a College of Medicine, University of Tennessee Health Science Center, 865 Jefferson Ave, Ste. F150, Memphis, TN 38163, USA; b Department of Radiology, University of Tennessee Health Science Center, 865 Jefferson Ave, Ste. F150, Memphis, TN 38163, USA

**Keywords:** HCC, TACE, Extrahepatic arterial supply

## Abstract

Hepatocellular carcinoma (HCC) is primarily supplied by hepatic arteries, but extrahepatic collaterals can occur. We present a rare case of HCC receiving blood supply from the right inferior adrenal artery (IAA). A 60-year-old male with HCC underwent multiple rounds of transarterial chemoembolization (TACE) for downstaging. After initial treatments, CT imaging revealed residual disease. Angiography demonstrated tumor supply from the right IAA. TACE was successfully performed via this vessel, leading to significant tumor necrosis confirmed by post-transplant pathology. This case emphasizes the importance of recognizing variant vascular supplies in HCC, especially in patients with prior TACE and tumors in segments VII and VIII. The IAA, while uncommon, should be considered as a possible collateral supply in challenging cases. Awareness of rare extrahepatic collateral supplies, such as the IAA, is crucial for achieving optimal outcomes in TACE procedures for HCC.

## Background

Hepatocellular Carcinoma (HCC) is the most common primary malignancy of the liver. For patients with unresectable HCC, transarterial chemoembolization (TACE) using drug-eluting beads is an established treatment, with clinical trials demonstrating significantly improved long-term survival [[1], [2], [3]]. TACE is a locoregional therapy causing both tissue ischemia and local chemotherapeutic drug delivery. Superselective TACE requires identification of tumor blood supply, thus knowledge of variation in standard vascular anatomy is necessary to achieve optimal treatment outcomes. While HCC is primarily vascularized by branches of the hepatic artery, blood supply from extrahepatic collateral vessels can be seen in approximately 17%-27% of patients [4]. Among them, the inferior phrenic artery (IPA) is most commonly involved in HCC with extrahepatic collateral arterial supply. Other common extrahepatic collateral supplies include omental arteries, intercostal arteries, internal mammary artery, right renal artery, and, occasionally, lumbar arteries [5,6]. Here, we present an unusual case of a 60-year-old male with HCC receiving aberrant blood supply from the right inferior adrenal (suprarenal) artery (IAA), who successfully underwent chemoembolization. While extrahepatic collateral supplies have been well-documented, involvement of the inferior adrenal artery is exceedingly rare.

## Case description

A 60-year-old Caucasian male was referred to our institution for management of HCC with the goal of downstaging to achieve liver transplantation eligibility. Initial computed tomography (CT) scan revealed 4 LI-RADS IV lesions in the right lobe of the liver measuring up to 4.5 cm, outside of transplant criteria. No evidence of extrahepatic disease or vascular invasion was observed. The extent of the patient's HCC initially exceeded both Milan and UCSF criteria for transplantation. Eastern Cooperative Oncology Group Performance Status (ECOG-PS) was 1. Child-Pugh score was 6, and Model for End Stage Liver Disease (MELD-Na) score ranged from 9-16.

**Fig. 1:**
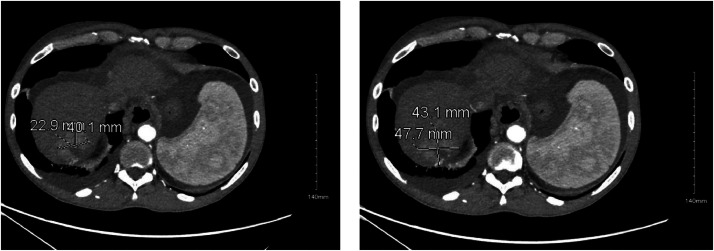
CT scan axial section, delayed phase. 2.3 cm x 4 cm lesion with early washout in Segment [VII] of liver.

After discussion at a multidisciplinary tumor board, the decision was made to treat with locoregional therapy to downstage the patient for transplant eligibility. The patient underwent transarterial chemoembolization (TACE) using 100-300 μm LC-beads and 75 mg doxorubicin to multiple branches supplying tumor of the right hepatic artery in July 2022. Follow-up CT scan 1 month after initial therapy showed trace residual disease in segment 8, leading to an additional TACE to the right inferior phrenic artery in September 2022. The initial follow-up scan at 1 month showed no evidence of disease. However, a subsequent study was shown to still have residual disease in segment 8. He then had a chemoembolization of the right hepatic artery in December 2022 at a different facility due to a change in insurance. Fig. 1 represents the CT imaging in February 2023 after 2 separate chemoembolizations of the right hepatic artery, demonstrating a right hepatic dome lesion compatible with residual viable tumor with peripheral enhancement having progressed since prior imaging.

**Fig. 2:**
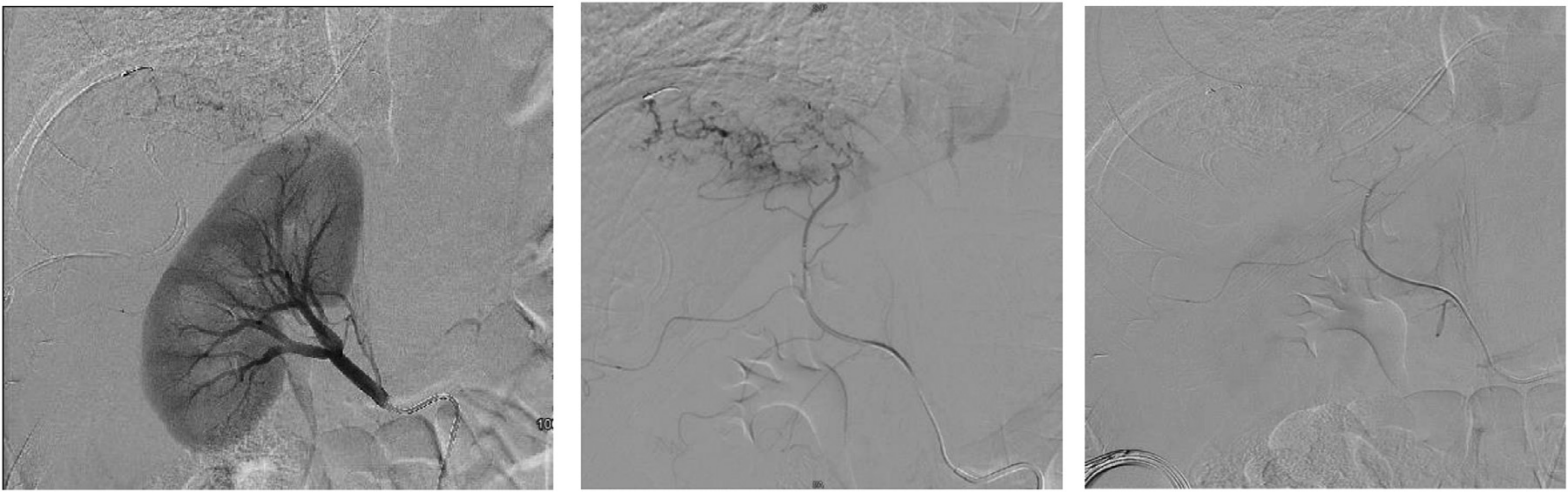
Selective catheter angiogram of inferior adrenal artery supplying the hepatocellular carcinoma with pre- and postembolization images.

His February 2023 CT scan showed disease progression with suspected supply from a branch originating from the right renal artery, necessitating another chemoembolization that was performed on April 17, 2023. Selective catheterization and arteriogram of the right IAA demonstrated tumor supply to the dome of the liver. TACE was performed via this vessel. Digital subtraction angiography (DSA) postembolization of the tumor branch of the IAA showed good adrenal perfusion. The patient was called for liver transplantation approximately 1 week after the most recent chemoembolization (April 23, 2023). His liver enzymes downtrended post-transplant and then remained stable. Following transplant, he recovered well and was discharged from the hospital on post-op day 5. Explant pathology showed > 85% necrosis of the hepatic disease. His allograft liver function has remained stable 6 months post-op Fig. 2.

## Discussion

The arterial supply of HCC tumors exhibits considerable variability. Approximately 55% of HCC is supplied solely by proper hepatic artery branches [6]. Cystic artery involvement accounts for 9% of HCC, often supplying those located near the gallbladder [5]. Among HCC with extrahepatic supply, adrenal arteries are rarely involved. A recent case reported HCC with lone arterial supply originating from the superior adrenal artery [7], which further highlights the degree of variation in HCC extrahepatic supply. In our case, the HCC received blood supply from the right inferior adrenal artery (IAA), which is a particularly uncommon source of extrahepatic collaterals. Interestingly, the IAA's origin, morphology, and course demonstrate the greatest variation among the 3 adrenal arteries. While the most common origin of IAA is the renal artery, it can branch off closer to the kidney, from a terminal branch of renal artery, or from a supplementary renal artery, resulting in variation of its relative position from other vessels. Other possible origins of IAA include aorta, gonadal artery, and celiac trunk [8,9], contributing to its unique courses.

The development of extrahepatic collateral vessels in HCC is often associated with specific tumor characteristics and treatment history. These risk factors include tumor size >5 cm [10,11], omental adhesion, and tumors located in segments VII and VIII, where they are in close proximity of extrahepatic structures [12]. Specifically, extrahepatic collateral supply can be a result of direct contact with other organs, including stomach, colon, kidney, and adrenal gland [13]. Other risk factors presented in patient history include history of TACE procedure [[14], [15], [16]]. Our patient notably had many of these risk factors including lesions in Segments VII and VIII and previous TACE procedures. Recognizing the potential for variant extrahepatic arterial supply in HCC is crucial for interventional radiologists performing TACE, particularly in challenging cases with residual disease after standard treatments. There are limited data on the efficacy of TACE in cases of extrahepatic tumor supply. This case report, therefore, offers a useful example of a rare deviation from the standard vascular supply to HCC, along with its treatment course and outcome.

## Patient consent

Informed consent for synthesis and publication of this case report was obtained from the patient.
